# Comment on Lee et al. Conservative Treatment with Teriparatide Versus Vertebroplasty for Acute Osteoporotic Vertebral Compression Fractures: A Meta-Analysis. *J. Clin. Med.* 2025, *14*, 3967

**DOI:** 10.3390/jcm15124694

**Published:** 2026-06-17

**Authors:** In-Suk Bae

**Affiliations:** Department of Neurosurgery, Nowon Eulji Medical Center, Eulji University, 68, Hangeulbiseok-ro, Nowon-gu, Seoul 01830, Republic of Korea; tbgitw@eulji.ac.kr

We read with great interest the article by Lee et al., titled “Conservative Treatment with Teriparatide Versus Vertebroplasty for Acute Osteoporotic Vertebral Compression Fractures: A Meta-Analysis” [1]. We would like to congratulate the authors for conducting the first meta-analysis comparing teriparatide (TP) conservative treatment with vertebroplasty (VP) for acute osteoporotic vertebral compression fractures (OVCFs). The authors concluded that TP offers long-term benefits in pain reduction, bone mineral density (BMD) improvement, and fracture prevention compared with VP.

VP has been widely performed to stabilize fractured vertebrae and provide immediate pain relief, particularly when conservative treatment has limitations [2]. However, high- to moderate-quality evidence indicates that VP does not yield significant clinical benefits compared with a sham procedure [3,4]. Cochrane reviews similarly do not recommend routine VP use due to its lack of superiority over placebo [5]. While therapeutic benefits of TP in OVCF-related nonunion have been reported [6], evidence evaluating its effectiveness in acute OVCF remains limited, making this meta-analysis highly meaningful.

This study follows PRISMA guidelines and PROSPERO registration, ensuring transparency and methodological rigor. Risk of bias was carefully assessed using RoB-2 for the single RCT and ROBINS-I for non-RCTs, with overall low risk across 75% of domains. Heterogeneity sources, including age and co-medications, were thoroughly explored via subgroup and sensitivity analyses, which strengthened the reliability of the pooled results. There was no significant difference in visual analog scale (VAS) score reduction between the two groups from one to three months. However, at six months, TP demonstrated clear superiority in both VAS and BMD outcomes. Furthermore, TP significantly reduced new-onset OVCFs, with an odds ratio of 0.15 (95% CI, 0.04–0.51, *p* < 0.01).

Several issues, however, merit consideration. Only five studies (one RCT and four non-RCTs; n = 326) were included, limiting statistical power. Most studies were observational, leading to moderate risks of confounding and bias. High heterogeneity in VAS results (I^2^ > 95% at most time points) weakens pooled estimates, despite partial resolution through subgroup analysis. Notably, kyphoplasty (KP), another widely used cement augmentation procedure for acute OVCFs, was excluded, reducing the study’s generalizability to all cement augmentation techniques [2,7]. In addition, variability in conservative protocols (e.g., analgesics, anti-resorptives) introduces unaddressed confounding factors. Although limitations such as small sample size, heterogeneity, and lack of standardization were discussed, issues of cost-effectiveness and patient compliance with daily TP injections and lifetime dosing limits warrant further attention.

Furthermore, a previous study reported greater progression of vertebral body collapse and wedge angle in the TP group compared with VP [8]. In the present meta-analysis, kyphotic angle and anterior height changes were evaluated, but only two studies were included, with follow-ups ranging from 3 to 18 months, possibly contributing to variability in outcomes and incident new-onset vertebral fracture rates.

Overall, we commend the authors for their comprehensive and methodologically robust analysis comparing TP and VP for acute OVCFs. We recommend future large-scale multicenter RCTs incorporating standardized treatment protocols and inclusion of KP for broader clinical relevance. Further studies should also evaluate TP’s cost-effectiveness and patient compliance challenges. As spine surgeons, we recognize TP as a valuable option for high-risk OVCF patients seeking to avoid cement-related complications such as leakage and adjacent segment fractures.

## Abbreviations

The following abbreviations are used in this manuscript:

TP	Teriparatide
VP	Vertebroplasty
OVCF	Osteoporotic vertebral compression fracture
BMD	Bone mineral density
VAS	Visual analog scale
KP	Kyphoplasty
